# My Second Thoughts About Brow Lifts: A Video Interview With Foad Nahai, MD, FACS

**DOI:** 10.1093/asjof/ojad092

**Published:** 2023-10-17

**Authors:** Foad Nahai, Lorne King Rosenfield

Welcome to another edition of Second Thoughts on First Thoughts. Today, we are honored to feature a colleague from whom the expression “needs no introduction” was inspired: Dr Foad Nahai (Video). In this conversation, you will go for a ride on Dr Nahai's brow lift learning curve—all the ups and downs as his strategies evolved from the coronal procedure to the endoscopic era to the direct temporal, upper eyelid, and glabellar approaches presently. With a surgeon boasting one of the most “mature” practices around, you are sure to glean a pearl or 2, as I did. So sit back and enjoy. And, thanks for watching!

## Supplementary Material

ojad092_Supplementary_Data

